# Successful eradication of *Mycoplasma hyopneumoniae* from the Norwegian pig population – 10 years later

**DOI:** 10.1186/s40813-021-00216-z

**Published:** 2021-05-17

**Authors:** Stine Margrethe Gulliksen, Børge Baustad, Tore Framstad, Anne Jørgensen, Audun Skomsøy, Oddbjørn Kjelvik, Mona Gjestvang, Carl Andreas Grøntvedt, Bjørn Lium

**Affiliations:** 1 Norwegian Pig Health Service, Animalia AS, P.O. Box 396, Økern, 0513 Oslo, Norway; 2 Department of Production Animal Clinical Sciences, Faculty of Veterinary Medicine and Biosciences, Norwegian University of Life Sciences (NMBU), P.O. Box 8146, Dep., 0033 Oslo, Norway; 3 Nortura SA, P.O. Box 360, Økern, 0513 Oslo, Norway; 4 The Meat and Poultry Industry National Organisation (KLF), Østensjøveien 39/41, N-0667 Oslo, Norway; 5 Norwegian Veterinary Institute, P.O. Box 750, Sentrum, 0106 Oslo, Norway

**Keywords:** Respiratory disease, Swine, Eradication, Program, Mycoplasma hyopneumoniae

## Abstract

**Background:**

*Mycoplasma hyopneumoniae* (Mhyo) is the causative agent of enzootic pneumonia in pigs which adversely affects animal health and welfare, in addition to causing considerable economical losses. This paper presents the implementation of the national Mhyo eradication program in Norway, the subsequent population wide surveillance and documentation on the current freedom from Mhyo in the Norwegian pig population.

In 1994, the Board of The Norwegian Pig Health Service decided on conducting a national surveillance and eradication program for Mhyo. The program aimed for population wide freedom from Mhyo, based on serological surveillance. A partial depopulation program was initiated in all Mhyo positive farrow-to-feed and farrow-to-finish herds. Total depopulation was performed in all positive finisher herds.

**Results:**

From 1994 to 2009, a total of 138,635 pigs in 3211 herds were serologically tested for the presence of antibodies against Mhyo. Of these, 5538 (4%) individual samples and 398 (12.4%) of the herds were defined as positive. In 2009, the Norwegian pig population was declared free from Mhyo, and has been so since then. From 2009 through 2019, a total of 44,228 individual serum samples have been analyzed for the presence of antibodies against Mhyo and found negative in the National surveillance program.

**Conclusion:**

Eradication of Mhyo infections has resulted in improved health and welfare of the Norwegian pig population. The success of the strategy is based on numerous factors, such as moderate to low prevalence of the agent, well documented and effective eradication protocols, accurate diagnostic tests, relatively small herds, low herd density in most parts of the country and negligible import of live pigs. In addition, economic benefit due to a premium on pigs marketed from herds free from Mhyo, a well-structured commercial pig population, and finally, the loyalty and significant effort of farmers, abattoir employees and veterinarians were crucial factors. To maintain the infection-free status at national level, a continuous alertness is required in the future to discover possible Mhyo infections and ensure rapid sampling and diagnostics. Any findings of Mhyo positive pig herds in Norway will result in immediate eradication.

## Background

*Mycoplasma hyopneumoniae* (Mhyo) is the causative agent of enzootic pneumonia (EP) in pigs and is prevalent in pig populations worldwide [28]. EP is adversely affecting animal welfare and is a significant cause of economic loss to pig producers due to decreased performance of the pigs and increased use of antibiotics [28, 35]. Mhyo has also been identified as an important contributor to the development and severity of porcine respiratory disease complex (PRDC), where it interacts with other pathogens, such as *Pasteurella multocida* (PMT), *Actinobacillus pleuropneumoniae* (APP), porcine reproductive and respiratory syndrome virus (PRRSV), swine influenza virus (SIV), and porcine circovirus type 2 (PCV2) [28].

The main clinical sign associated with Mhyo infections is a chronic non-productive cough appearing 10 to 16 days post infection [38], and is usually evident in grower-finishing pigs. Mhyo disrupts the respiratory mucosal clearance system by colonizing the cilia on the epithelial surface, and by modulating the immune system of the respiratory tract [30]. The pathogen is mainly transmitted horizontally from infected pigs to non-infected pen mates and from sows to their offspring during the suckling period [25, 26, 31].

In order to improve animal welfare and reduce the economic losses, several control measures, including optimizing management and housing, vaccination and strategic antimicrobial medication are utilized (reviewed by [12, 22]). When preventive measures are insufficient, or when herd freedom from Mhyo is desired, eradication protocols may be implemented [12]. Various eradication protocols have been described and are well established in many countries, including depopulation and repopulation, partial depopulation, herd closure (i.e. ceasing introduction of replacement sows into the herd for at least 6 months) and medication [12], and whole-herd medication without herd closure [40]. A regional, and eventually, national eradication program was first implemented in Switzerland in the late 1980s [34, 41]. The Swiss program aimed to obtain farms free from clinical disease caused by Mhyo and defined serotypes of APP based on a method including partial depopulation. A similar program was implemented in Finland a few years later [10, 29].

In the 1990s, lung lesions indicative of Mhyo infections were found in 10 to 20% of Norwegian slaughter pigs scored at the abattoir [3, 15, 17, 18], and preliminary serological surveys of the Norwegian pig population indicated that between 15 and 20% of the sow herds were infected with Mhyo, however with a substantial variation between counties [1, 13].

In 1994, the Norwegian pig production sector (including The Norwegian Pig Health Service, the pig breeder’s organization (Norsvin SA) and the abattoir organizations) agreed on a long-term strategy for surveillance and control of EP. The strategy included the ambition of implementing a national eradication program for Mhyo with the goal of population-wide freedom. The intermediate objectives were to maintain the Mhyo-free status of all nucleus and multiplier breeding herds and to perform a serological screening for antibodies against Mhyo in all sow herds. In addition, the strategy aimed for strictly regulated trade and thereby preventing pigs from infected herds being mixed with pigs from non-infected herds. The final objectives were to eradicate Mhyo from all pig herds in defined regions in 2–3 years, and from all herds in the country within 5 to 8 years.

This paper presents the implementation of the national eradication program in Norway, the subsequent population wide surveillance and documentation on the current freedom from Mhyo in the Norwegian pig population.

## Results

**Fig. 1:**
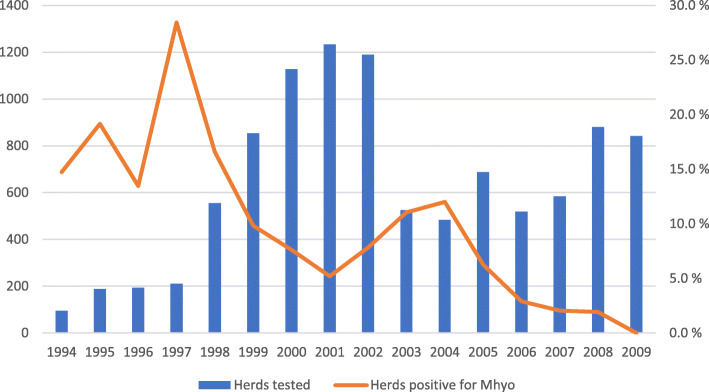
Number of Norwegian pig herds tested per year, and the proportion of Mhyo positive herds. Legend: Number of Norwegian pig herds tested per year between 1994 and 2009, and the proportion of herds positive for antibodies against *Mycoplasma hyopneumoniae* (Mhyo). Most herds were tested more than once

Between 1994 and 2009, serum or colostrum samples from a total of 138,635 pigs in 3211 herds were serologically tested for the presence of antibodies against Mhyo (Table 1). Of these, 5538 (4%) individual samples and 398 (12.4%) of the herds were defined as positive. The screening detected no positive herds in the counties of Telemark, and Sogn and Fjordane. The highest proportion of positive herds (19.7%) were found in Rogaland county in the south-west of Norway. Number of herds tested yearly ranged from 95 (in 1994) to 1233 (in 2001), and most herds were tested more than once during the period (Fig. 1).

**Table 1:** Results from the Mhyo screening of serum and colostrum samples from Norwegian pig herds

County	Herds tested	Herds Mhyo positive (%)	Last year of Mhyo positive sample
**Østfold**	192	13 (6.8)	2003
**Akershus**	135	17 (12.6)	2005
**Hedmark**	257	33 (12.8)	2005
**Oppland**	253	16 (6.3)	2006
**Buskerud**	42	4 (9.5)	2005
**Vestfold**	160	10 (6.3)	2003
**Telemark**	44	0 (0)	
**Aust-Agder**	17	3 (17.6)	1999
**Vest-Agder**	29	2 (6.9)	2006
**Rogaland**	988	195 (19.7)	2008
**Hordaland**	106	6 (5.7)	2007
**Sogn- og Fjordane**	83	0 (0)	
**Møre og Romsdal**	93	14 (14.3)	2003
**Sør-Trøndelag**	124	17 (13.7)	2005
**Nord-Trøndelag**	523	57 (10.9)	2005
**Nordland**	114	9 (7.9)	2001
**Troms**	38	1 (2.6)	2002
**Finnmark**	13	1 (7.6)	2001
**Norway**	**3211**	**398 (12.4)**	**2008**

*Legend*: Results from the screening of serum and colostrum samples from Norwegian pig herds serologically tested for the presence of antibodies against *Mycoplasma hyopneumoniae * (Mhyo) between 1994 and 2008

Between 1994 and 1998 all nucleus and multiplier herds (*n* = 216) were tested for antibodies against Mhyo. A total of 12.5% of these (*n* = 27) were found positive and were obliged to perform an eradication program. In 1998 the annual serological screening for antibodies to Mhyo detected no seropositive nucleus herds and only one seropositive multiplier herd. During the years 1999 to 2003 all nucleus and multiplier herds were documented free from Mhyo. One multiplier herd in Rogaland county was reinfected by Mhyo both in 2004 and 2007, most probably from contaminated animal transport vehicles or infected neighboring herds. All nucleus and multiplier herds have been free from Mhyo since 2007.

By the end of 2003 and 2005 the pig population in nine and fourteen of 18 counties respectively, were considered free from Mhyo (Table 1). The last known Mhyo positive sow herd in Rogaland completed the eradication program in October 2006, and by the end of the year, there were no known Mhyo positive sow herds in Norway.

In 2007 an intensive serological screening of all sow herds in Rogaland (*n* = 390) was performed. Seven of the 390 sow herds (1.8%) were found positive. In addition, in October, the multiplier herd, mentioned above, tested positive again. The infection had spread to five commercial herds through trade of live pigs from the multiplier herd. The cause of the reinfection was never discovered. Based on this finding, it was decided to test all pig herds in Rogaland during 2008 and 2009, both sow herds and finisher herds. Due to high herd density and a history of several reinfections in this area, commercial Mhyo vaccines (Respisure™ or Suvaxyn Mhyo™) were used in Mhyo positive sow herds (*n* = 8) in Rogaland in 2006 to 2008.

In April 2008, a sow pool system, including the central insemination and gestation unit and six out of nine associated farrow-to-feed satellite herds all localized in Rogaland were found positive. In addition, five finisher herds associated with the sow pool system were found positive. In all these herds it was decided to perform eradication through total depopulation in order to quickly eliminate Mhyo positive pigs and holdings from the area. Subsequent washing and disinfection were performed before restocking with pigs from Mhyo-free herds. The last eradications were completed in 2008.

From 2009 through 2019, a total of 44,228 individual serum samples have been analyzed for the presence of antibodies against Mhyo (Table 2). From 2009 to 2015, all samples tested were negative. In 2016 and 2017, samples from two and one herd, respectively, were found positive. The samples were found in herds performing vaccination against Mhyo in relation to planned export of gilts.

**Table 2:** Results from serological testing of Norwegian pig herds for M*ycoplasma hyopneumoniae *(Mhyo)

Year	Herds	Samples	Herds with Mhyo antibodies detected
**2009**	847	9620	0
**2010**	212	2745	0
**2011**	210	2415	0
**2012**	777	5348	0
**2013**	763	5348	0
**2014**	467	3321	0
**2015**	398	2938	0
**2016**	458	2972	2^a^
**2017**	385	3021	1^b^
**2018**	362	3070	0
**2019**	397	3430	0

*Legend*: Results from serological testing of Norwegian pig herds for the occurrence of antibodies against *Mycoplasma hyopneumoniae *(Mhyo) in the national control program 2009–2019

^a^Six and four samples from two herds respectively, tested positive for the occurrence of antibodies against Mhyo due to vaccination of gilts for export

^b^Two samples from one herd identical to one of the two herds from 2016, tested positive for the occurrence of antibodies against Mhyo due to vaccination of gilts for export

Except for the eradications in the sow pool system in 2008, where much of the costs were paid by the insurance and slaughterhouse companies, the costs of the eradications were paid by the farmers. The costs varied widely between herds, depending on the production form, building facilities and the planning.

Approximately 40% of the analysis costs were covered by the farmers, while 40–50% were paid by the Norwegian Pig Health Service through collective funds from a pork levy all farmers are required to pay based on kilograms delivered to the abattoir. The last 10–20% were payed primarily by the abattoirs. In addition, Norsvin SA and The Norwegian Veterinary Institute contributed with smaller amounts. The costs per herd varied immensely depending on herd type, and size, and how the herds managed to organize the production flow during the eradication period.

The annual economic benefit of freedom from Mhyo in the Norwegian pig population (1.5 mill pigs slaughtered per year) was in 2009 roughly estimated to 7–18 million NOK [20].

## Discussion

### Comparison with other eradication programs

The benefits of disease-free populations of pigs include improved animal welfare, increased production at reduced cost, reduced need for antibiotics and increased job satisfaction. Several countries have established programs to create pig herds free from defined respiratory agents, such as Mhyo [12], but regional and national eradication and control measures for Mhyo have to our knowledge only been implemented in Switzerland [41], Finland [10] and Norway [19]. During the initial screenings in Switzerland in 1994, the annual incidence of EP in pig herds joining the Swiss Pig health Service (SPHS), accounting for 85% of the breeding and multiplying farms, was found to be 2–4%, with regional differences and local maximum incidences up to 12% [34]. This coincides well with the initial pre-campaign results from Norway, although the SPHS herds in Switzerland were already monitored for respiratory disease and classified as high health herds prior to the serological screening. In comparison, antibodies to Mhyo were detected in 65% of 2578 sows in 100% of 67 herds in north-west Germany [6]. The low incidences of Mhyo infection found in Norway, Finland and Switzerland have been important in order to succeed with the elimination protocols at regional and national level. Heinonen et al. [10] reported a success rate of 81% in their eradication program performed in 21 Mhyo infected pig herds joining a Finnish health class program. A similar success rate was found in the Norwegian eradication program, although the number of failed eradications varied between regions, with the highest occurrence of failures in Rogaland. These failures were mainly due to inadequate follow-up of the eradication protocols, reinfections from neighboring herds and infected animal transport vehicles.

In Switzerland, trade of subclinically infected live animals was found to cause most outbreaks of respiratory disease during the national control program [9, 34]. This coincides with the experience from Norway. In the Norwegian program, trade with live pigs was strictly regulated to prevent pigs from infected herds being mixed with pigs from non-infected herds, but in spite of this, most of the reinfections were probably caused by trade of infected pigs or spread by contaminated transport vehicles. Unfortunately, there was not performed any research to trace the source of reinfections.

### Eradication protocols

Total depopulation and repopulation (depop-repop) have long been recognized as the most successful and reliable means to eradicate disease [12]. In addition, it includes the possibility to eliminate more than one pathogen at once, and the opportunity to improve genetics. On the other hand, total depop-repop includes a complete loss of production from the time the herd is emptied until replacement sows begin farrowing, and it is undesirable on farms with animals of high genetic value (i.e. nucleus or multiplier farms). The partial depopulation protocol used in the Norwegian eradication program, is less expensive [41], but it is mainly suited for small herds [22]. In Rogaland, eradication through complete depop-repop was performed in all Mhyo positive herds in the final stage of the eradication program. Additional efforts were needed to succeed, and an integrated approach across the entire region was necessary to control the infection. There were several reasons for the additional challenges in this region, including high herd density, continuous production in several finisher herds and poorer regulation of trade in live pigs. The vaccination used in the last eight Mhyo positive sow herds in this county in the last years of the eradication program was an attempt to reduce the infectious pressure both in the herds and relative to neighboring herds during the period from the herds tested positive until the eradication program was performed. Vaccines have been found to reduce the number of Mhyo organisms in the respiratory tract and decrease the infection level in a herd [18, 24, 32]. The effect of implementing this measure in Rogaland at the end stage of the national eradication program is uncertain and was most probably not essential for the success of the program.

### Risk factors

Stärk et al. [33] found that the distance between the herds, the density of the pig population in the area, the distance to the road and differences in the topography were important risk factors for reinfection of herds with Mhyo. Norway, Switzerland and Finland have favorable geographical conditions with mountains and forests creating natural infection barriers and low herd densities, reducing the risk of reinfections. Several studies show that the health status of neighboring farms and their geographical distance from the farm under observation are significant risk factors for Mhyo infection [5, 33, 39]. Dee et al. [2] showed that Mhyo could be identified in samples 4.7 km from their source, and Otake et al. [27] recovered Mhyo in air samples more than 9 km from their source. In the Norwegian eradication program, the distance between pig herds was only 2–300 m in several cases. Despite this, the number of reinfections was low. Similar to Finland and Switzerland, the herd sizes in Norway are small in an international perspective. Large herd size has generally been considered a risk factor for respiratory disease in pigs [22], hence the low incidences of Mhyo infections and the low number of reinfections in these countries, might have been influenced by this. The risk of airborne transmission of pathogens is also reduced when the herd sizes are small and the number of susceptible animals in an area in general is low [33]. On the other hand, other studies have shown no association between herd size and the prevalence of Mhyo infections [6, 21], and even a reduced risk for disease in large herds has been reported, due to owners of large herds more frequently adopting management and housing practices that reduce this risk compared with owners of smaller herds [4].

Management factors such as production system, purchase of animals, animal stocking density, biosecurity measures and housing conditions are important factors in the control of respiratory infections [10]. All-in-all-out management of the farrowing units in sow herds and age segregated systems in herds with grower-finishers have been found beneficial as it can help prevent cross-contamination between batches and allows the farmer to clean the facilities between groups of pigs [6, 11]. Heinonen et al. [10] concluded that the distance between infected and uninfected pigs was one of the most important risk factors for the spreading of Mhyo and recommended that different age groups of animals always should be kept as far away from each other as possible to avoid airborne transmission. In Norway, all-in-all-out production, either by compartment in sow herds, or on herd level in finisher herds, was introduced in many herds during the eradication program. Also, other biosecurity measures within and between herds, i.e. daily cleaning routines and proper use of biosecurity barriers like hygiene locks for persons entering the pig holdings, were improved to reduce the risk of infection.

The commercial Norwegian pig population is organized in a pyramidal structure with unidirectional animal flow from closed pure-breed genetic nucleus herds at the apex, through multiplier herds producing hybrid sows for productions herds (farrow-to-feed or farrow-to-finish) and specialized fattening pig producers. Hence, by annual serological testing for Mhyo of all nucleus and multiplier herds and sow pool systems, the potential risk of spreading a possible infection is limited. The risk of reintroduction of Mhyo in the Norwegian pig population is relatively small as there are mandatory requirements of documented Mhyo freedom for all import of pigs from abroad, and the import itself is negligible. From 1994 to 2018 a total of 254 pigs in 11 batches were imported, only 36 of them (in two imports) after 2008 [36]. Continued systematic testing of all imported live pigs is crucial to reduce the risk of reinfection to the pig population.

The growing interest for outdoor pig production represents an increased concern of introducing Mhyo through direct contact between potentially infected wild boar and domesticated pigs kept outdoor. Therefore, in 2018 the Norwegian Veterinary Institute, in cooperation with the Norwegian pig production sector, initiated a surveillance program for Mhyo in wild boar. During 2018 and 2019, blood samples from a total of 92 wild boars were tested for the occurrence of antibodies against Mhyo. All samples were negative [8]. The results of a Swiss study [14] concluded that transmission of Mhyo between wild boars and domestic pigs is possible, but the persistence of Mhyo within a farm as well as transmission between farms were factors more important for sporadic outbreaks than contact to wild boar. Further surveillance is warranted to consider the potential significance of wild boar as a risk factor for introduction of Mhyo to the Norwegian domestic pig population.

Another potential risk factor is the use of mini pigs as companion animals. Mini pigs are not part of the surveillance program for Mhyo in pigs in Norway. Even though mini pigs are normally kept as pets, and as such away from pigs in commercial farms, there is still a risk of spreading pathogens between the two. This is exemplified by some farms having used mini pigs as teaser boars for stimulating the sows before artificial insemination. After entering the breeding herd, these teaser boars usually come in close nose-to-nose contact with a considerable portion of the sow herd within a short time period. The use of mini pigs as teaser boars is not in accordance with the Norwegian pig productions sector’s recommendations.

### Diagnostic tests

When tracking swine respiratory disease outbreaks in endemically Mhyo infected populations, various diagnostic methods are available [23], but serological testing still seems to be the preferred method to document population freedom. As opposed to the Swiss Mhyo surveillance- and control program aiming to maintain the absence of disease [34], the goal of the Norwegian Mhyo program is the documentation of the absence of the causative agent. Like Norway, Finland used serological mass testing for this purpose after completion of the eradication program [29]. Serological analysis is a rapid and inexpensive method for screening of pathogens, and the sensitivity and specificity of the ELISA test used both in Norway and Finland is extremely high and have been reported to be 100% (98–100%, confidence interval 95%) and 100% (93–100%), respectively [37]. In these tests, antibodies are detected regardless of origin. Hence, antibodies acquired through maternal immunity or immunization through vaccination can result in seropositivity [22]. In Norway in 2016 and 2017, positive samples were found in two herds and one herd, respectively. Retesting of these herds was planned, and all transport of pigs from the seropositive herds was immediately stopped. But when going through the identification numbers of the sampled pigs, it was discovered that the seropositive individual pigs had been vaccinated due to planned exports of gilts in the nucleus herd from which the pigs originated. Hence, the additional sampling was cancelled. All samples from these herds have been negative since. Improved routines of vaccination records in the nucleus herds were implemented to avoid similar incidences in the future. It would be beneficial, and research is needed, to further improve Mhyo ELISAs to include discrimination between infected and vaccinated pigs [23]. However, in Norway, in a highly regulated and non-vaccinated population, this challenge is greatly reduced or eliminated.

## Conclusions

The success of the Norwegian eradication strategy is based on ambitious decisions made by the board of the Norwegian Pig Health Service, but several factors have been crucial to obtain and are still crucial to maintain the national status of Mhyo freedom. This includes the initial moderate to low incidence of the agent, well documented and effective eradication protocols, accurate diagnostic tests, small commercial herds in an international perspective, low herd density and negligible import of live pigs with mandatory requirements of documented Mhyo freedom for those imported. In addition, a well-structured commercial pig population with stringent adherence to unidirectional live animal flow, the economic benefit due to a premium on pigs marketed from herds free from Mhyo, and finally, the loyalty and significant effort of farmers, abattoir employees and local veterinarians were crucial factors for success.

The prolonged active serosurveillance, in combination with the low prevalence of EP-like lesions in abattoirs, provides a solid basis for concluding that the commercial pig population in Norway is free from Mhyo. To maintain the infection-free status at national level, a continuous alertness from veterinarians, the food safety authorities performing clinical registrations at the abattoir, farmers and other advisors is required in the future to discover possible Mhyo infections and ensure rapid sampling and diagnostics. Any findings of Mhyo positive pig herds in Norway will result in immediate eradication.

## Methods

### Initial eradication attempt

Already in 1992, four farrow-to-finish herds (two nucleus herds and two farrow-to-finish herds), with an average size of 25 sows, were selected for an Mhyo eradication attempt [16]. The diagnosis of EP was based on clinical signs (coughing and/or dyspnea), serology (ELISA), an indirect immunofluorescence (IIF) assay and pathological findings in lungs indicative of Mhyo infection (chronic consolidation and/or pleuritis of the cranioventral lung) [16]. Clinical signs of respiratory disease were found in pigs in all four herds, the prevalence of antibodies against Mhyo was found to be 54–100%, and the incidence of lung lesions at slaughter ranged from 23.6 to 40% in the participating herds. Lesions at slaughter were recorded according to defined protocols from the Norwegian Food Safety Authority at that time. The eradications were carried out according to the method described by Zimmerman et al. [41], except that suckling piglets were present during the eradication period in two of the herds. All units and pens were cleaned and disinfected, using sodium hypochlorite and calcium hydroxide as disinfectants.

To control the Mhyo status of each herd, fatteners born after completing the eradication were serologically tested at slaughter (30–113 samples per herd). In addition, lungs from all pigs slaughtered were examined macroscopically for lesions 18–33 months after the eradication program was completed. Lungs with lesions suggestive of Mhyo infection were subject to histopathological examination.

All sampled fatteners in participating herds were seronegative after the eradications, and the average prevalence of gross lesions indicative of pneumonia and pleurisy recorded at slaughter in the four herds was reduced from 34.3 to 2.5 and 7.4 to 2.4%, respectively [16].

Loss of production was minimal in all 4 units, as young animals (growers, finishers) were transferred to other units. In two herds (the nucleus herds) the average daily weight gain (ADG) from birth to 138 days of age increased by 92 and 33 g respectively after eradication compared to before. In the two other herds the period from birth to slaughter was reduced by 1 to 1.5 months.

### From initial attempt to national program

Several important events led to the implementation of a screening and eradication program for Mhyo on a national level (Table 3). Based on the promising results from the initial eradication attempt, all nucleus and multiplier breeding herds were tested for the presence of antibodies to Mhyo between 1994 and 1997. Positive herds had to perform an eradication program. In 1997 the screening was extended to include commercial sow herds, as the farmer-owned abattoirs initiated a project for trading high-health feeder piglets. This project included a serological screening of sow herds for the occurrence of antibodies against Mhyo, in addition to systematic clinical inspections for progressive atrophic rhinitis (caused by toxin producing *Pasteurella multocida* (PMT)), sarcoptic mange and swine dysentery (caused by *Brachyspira hyodysenteriae*) [13]. In 1999, a premium of 14% was introduced for high-health feeder piglets documented free from Mhyo, in both private end farmer-owned abbatoirs (Table 3). Based on the results of the screening, regional eradication of Mhyo was initiated in counties with low herd prevalence. In 2000, a national serological screening and eradication program for Mhyo in all commercial pig herds was initiated. The aim was national freedom from Mhyo in the pig population by the end of 2005.

**Table 3:** Events in the national screening and eradication program for *Mycoplasma hyopneumoniae *(Mhyo in the Norwegian pig population

Year	Important events
**1992**	Initial eradication attempts successfully performed in four farrow-to-finnish herds
**1994–1997**	Screening and eradication of Mhyo in nucleus and multiplier herds
**1997**	The farmer-owned abattoirs initiate a project for trading high-health feeder piglets.
**1998**	All nucleus and multiplier herds free from Mhyo by the end of the year. Regional eradication of Mhyo in commercial herds is initiated in counties with low herd prevalence of Mhyo (Vestfold, Telemark, Buskerud and Trøndelag).
**1999**	A premium of 14% is introduced for high-health feeder piglets documented free from Mhyo, toxinproducing *Pasteurella multocida*, *Sarcoptes scabiei* and *Brachyspira hyodysenteriae* in both private end farmer-owned abbatoirs.
**2000**	National eradication program with the aim of freedom from Mhyo in the entire pig population by the end of 2005 is decided.
**2001**	Action plan with the aim of known Mhyo status in all sow herds before the end of 2002 is decided.
**2002**	Mycoplasma project Rogaland is initiated.
**2004**	All herds in 9 of 18 counties free from Mhyo (Table 1) Large multiplier herd in Rogaland reinfected.
**2006**	A deduction of 100 NOK per finisher pig slaughtered from herds not yet declared free from Mhyo is implemented by all abattoirs. All sow herds tested free from Mhyo by the end of the year.
**2007**	Retesting of all sow herds in Rogaland county. October: New reinfection in the multiplier herd in Rogaland including five contact herds.
**2008**	Retesting of all pig herds in Rogaland. April: A sow pool system tested positive, eradication performed by total depopulation and repopulated with pigs from herds documented free from Mhyo
**2009**	First year without any Mhyo positive samples National surveillance program for Mhyo is initiated

*Legend*: Important events in the development, implementation and the final success of a national screening and eradication program for *Mycoplasma hyopneumoniae *(Mhyo) in the Norwegian pig population

The Norwegian Pig Health Service was responsible for the planning and implementation of the project, but the eradication program was primarily based on voluntary efforts from farmers and was to be a joint effort of private and farmer owned abattoirs, and Norsvin. All costs, including laboratory analyses, were paid by the pig production sector, shared in part by the affected farmers individually and in part through collective funds from a pork levy all farmers are required to pay based on kilograms delivered to the abattoir.

Due to higher herd density and a higher prevalence of Mhyo positive herds in Rogaland county compared to the rest of the country, additional efforts were necessary to succeed with the eradication program here. Therefore, in 2002, a separate project group for Mhyo eradication in Rogaland was established. The project group was comprised by representatives from the Norwegian Pig Health Service, the local/regional abattoirs and the pig breeder’s organization (Norsvin SA).

### Screening and eradication

In nucleus and multiplier breeding herds, blood samples from 60 and 40 conveniently sampled fatteners or sows from each herd, respectively were collected [19]. In other herd categories, at least 20 blood samples from fatteners or 20 blood- or colostrum samples from sows were collected [20]. The samples were analyzed by a monoclonal blocking enzyme-linked immunosorbent assay (ELISA; *Mycoplasma hyopneumoniae* ELISA, DAKO®, Denmark) according to the manufacturers’ instructions [37]. All analyses were performed at the Norwegian Veterinary Institute.

If all samples were found negative, the herd was classified as free from Mhyo. All herds with three or more positive samples were defined as positive. In herds with one or two positive samples, new samples were collected, and the herd was retested before the conclusion on Mhyo status was made.

All herds defined as positive for Mhyo, were contacted by an advisor or veterinarian from their abattoir to plan the eradication, at least 6 months before scheduled startup. Each eradication was thoroughly planned to minimize the production losses during the eradication period. Before eradication was initiated, the risk of reinfection from neighboring farms or transport vehicles was considered. The inseminations were planned to avoid farrowing in a period of at least 4, and preferably 6 weeks.

A partial depopulation program according to the Swiss method [41], was initiated in Mhyo positive sow herds. All pigs under the age of 10 months (suckling piglets, weaners, growers and fatteners) were removed from the infected herds. For a period of 14 days, only breeding animals (sows and boars) of more than 10 months of age, clinically free from EP were present in the herd. All breeding animals were medicated with tiamulin (then Tiamutin™, now Denagard™, Elanco) with 120 ppm (10 ml Tiamutin™, 125 mg/ml per 10 l/water), a dose equivalent to 6 to10 mg tiamulin/kg bodyweight for 14 days through the drinking water or by feeding a medicated liquid feed [16]. Pigs getting ill during the eradication period were either removed from the herd or treated with Tiamutin™ injectable (1 ml per 20 kg BW).

All units and pens were cleaned and disinfected, using sodium hypochlorite or potassium peroxymonosulfate (Virkon S®) as disinfectants. Cleaning and disinfection were preferably performed in empty units. The herds were restocked by the medicated sows and piglets born after completed eradication. In the great majority of positive herds, the eradication was performed according to this protocol, but in a few farms suckling piglets younger than 3 weeks of age were present during the eradication period. Also, in some of the Mhyo positive herds the farmer decided to discontinue the pig production. In Mhyo positive finisher herds, total depopulation by slaughtering finished pigs was performed, followed by cleaning and disinfection as described above.

To confirm freedom from Mhyo after eradication, all herds were retested by analyzing blood or colostrum samples from 10 pigs and/or sows twice a year, in total 20 samples per herd. Additional sampling was performed in herds with clinical symptoms of respiratory disease and in herds where the prevalence of gross lesions indicative of pneumonia and pleurisy recorded at slaughter was high.

### Surveillance program

Since 1997, all nucleus and multiplier herds have been tested for the occurrence of antibodies against Mhyo, based on blood or colostrum samples from at least ten pigs in each herd twice a year [19]. The number of nucleus- and multiplier herds varies from year to year and have been reduced from 194 in 1997 to 80 in 2019. Pigs in these herds are also clinically controlled for signs of respiratory disease at least three times per year by the herd veterinarian. All recordings from the visits are reported through a digital health recording system owned by the abattoirs.

Since 2009, all sow pool systems and samples collected from other herd categories (integrated herds and piglet-producing herds) have also been analyzed for antibodies against Mhyo. The testing performed until 2011 was organized by the Norwegian Pig Health Service. From 2012, the samples have been collected through the Surveillance Program for Specific Viral Infections in Swine Herds in Norway, implemented by the Norwegian Food Safety Authority (NFSA) [7]. Plucks from all slaughtered pigs are screened by personnel from the NFSA for gross lesions indicative of pneumonia and pleurisy at slaughter. Observations of lesions indicating Mhyo infection are immediately followed up by serological examinations in the herds.

Serosurveillance for Mhyo is financed by the Norwegian Pig Health Service and analyses are performed by the Norwegian Veterinary Institute by a monoclonal blocking ELISA, as described above.

## Data Availability

The datasets used during the current study are available in an anonymized form from the corresponding author on reasonable request.
